# Rare Constellation of Pelvic Injuries: A Case Report

**DOI:** 10.7759/cureus.23077

**Published:** 2022-03-11

**Authors:** Ghadeer Alsager, Hasan Aleisawi, Hussain Alyousif, Hani Alsarhan

**Affiliations:** 1 Orthopaedic Surgery, King Saud University Medical City, Riyadh, SAU; 2 Orthopaedic Surgery, King Saud Medical City, Riyadh, SAU

**Keywords:** morel-lavallée lesion, mortality, open pelvic fracture, bilateral crescent fracture dislocation, degloving injury, common iliac artery injury

## Abstract

Crescent fracture-dislocations are sunset of lateral compression injuries. They can be associated with vascular, bowel, genitourinary, or soft tissue degloving injuries. Here, we describe a patient with bilateral crescent fracture-dislocation, right common iliac artery (CIA) injury, and an extensive Morel-Lavallée lesion (MLL). A 35-year-old male was transferred to our hospital after being involved in a motor vehicle collision with an unknown mechanism. Upon evaluation, four rare injuries were found: right CIA injury, bilateral open iliac bone fracture, bilateral crescent fracture-dislocation, and an extensive MLL. The patient underwent successful right CIA thrombectomy and stenting, followed by irrigation and debridement of MLL and open pelvic fractures. However, his hospital course was complicated by septic shock with spontaneous rectal perforation, necessitating massive transfusion protocol activation and three relook laparotomies. Unfortunately, the patient died after 25 days of a turbulent hospital course. The combination of bilateral crescent fracture-dislocation, bilateral open pelvic fracture, CIA injury, and an extensive MLL is exceedingly rare. Each poses a challenge when encountered alone, nevertheless, after establishing hemodynamic stability, timely intervention is crucial to avoid possible morbidity and mortality.

## Introduction

Crescent fracture-dislocation compromises only 12% of pelvic ring fractures [1]. The association of pelvic ring fracture with other injuries is well documented. Among those, Morel-Lavallée lesion (MLL) has been linked to such injuries. MLLs are degloving injuries that tend to appear around the greater trochanter (30.4%), followed by the thigh (20.1%) and pelvis (18.6%), with an average size of 30 × 12 cm [2,3]. Among other associated injuries with pelvic ring fractures are vascular injuries. The internal iliac artery and its branches, especially the superior gluteal artery, is the most commonly injured vessel in pelvic trauma [4]. However, blunt injuries to the common iliac artery (CIA) are extremely rare. We describe an unusual but unfortunate coincidence of a triad of rare injuries that have never been in unity, let alone combined in one human being. To our knowledge, this is the first case report combining bilateral crescent fracture-dislocation, CIA injury, bilateral open iliac fracture, and an extensive MLL. Management and complications are discussed along with a detailed description of the case.

## Case presentation

**Figure 1:**
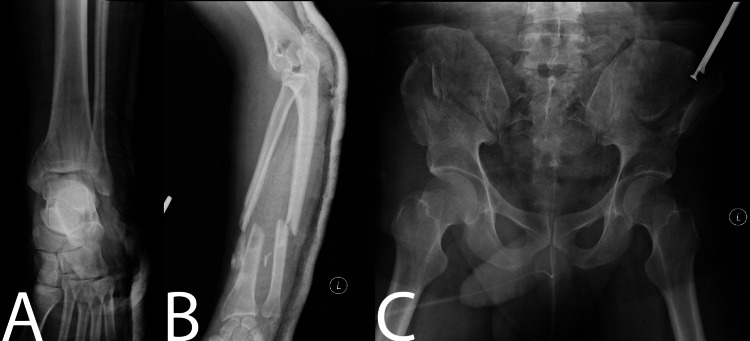
Skeletal survey radiographs. Radiographs showing (A) left medial malleolus fracture, (B) left forearm fracture, (C) bilateral iliac wing fractures as well as bilateral crescent fracture-dislocation extending to the sacroiliac joint.

**Figure 2:**
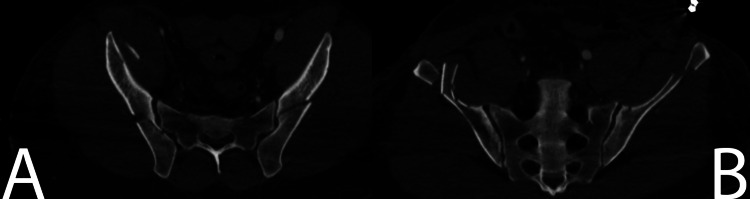
Reformatted CT images of the posterior pelvic ring. (A) Inlet reformatted CT image of the posterior pelvic ring demonstrating bilateral fracture lines entering the sacroiliac joint. (B) Outlet reformatted CT image of the posterior pelvic ring demonstrating diastasis of the upper sacroiliac joint consistent with crescent fracture-dislocation. CT: computed tomography

**Figure 3:**
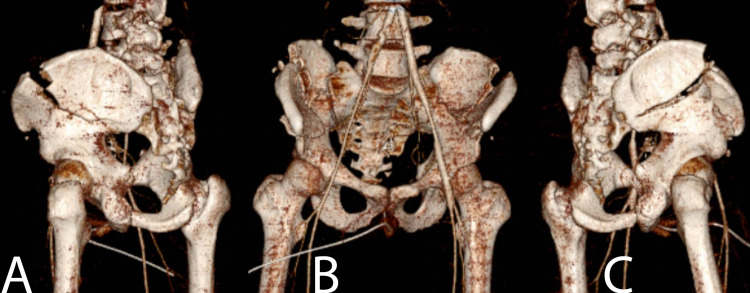
3D reformatted CT images of the pelvis. (A) 3D reformatted CT image demonstrating left crescent fracture as well as iliac wing fracture. (B) 3D reformatted CT image demonstrating bilateral crescent fracture-dislocation as well as bilateral iliac wing fractures. (C) 3D reformatted CT image demonstrating right crescent fracture as well as iliac wing fracture. 3D: three-dimensional; CT: computed tomography

A 35-year-old male with an unknown medical history was referred to our hospital as a life-saving polytrauma case after being involved in a motor vehicle collision. Initially, the patient was resuscitated, intubated due to a low Glasgow Coma Scale (GCS) score of 7/15 at a peripheral hospital, and a right intercostal tube (ICT) was placed. Upon arrival to our hospital, the patient was re-evaluated by the emergency and trauma teams according to the advanced trauma life support (ATLS®) protocol. The patient generally looked ill with a low blood pressure (BP) of 99/63 mmHg. He was mechanically ventilated with volume control-assist control (VC-AC) mode and on a cervical collar. The chest wall showed symmetrical rising and blood drainage through the ICT. There was no obvious external bleeding. A pelvic sheet was wrapped around the pelvis which was removed. Complete exposure was performed which showed multiple scattered lacerations and abrasions, with a 5 mm puncture wound on the volar left forearm and bilateral wounds of 8 cm over the iliac crests. These wounds were sutured at the peripheral hospital. Examination of the distal extremities revealed cold and bluish discoloration of the right lower extremity. Capillary refill was significantly delayed (5.5 seconds) and pulses were not palpable. GCS score was 7/15 (E1V1M5), with 2 mm reactive pupils. There was an extensive ecchymosis over the lower abdomen extending to the upper thighs and flanks. Arterial blood gas analysis was done (Table 1). Extended focused assessment with sonography for trauma failed to show any lung slide on the right chest which confirmed the diagnosis of right hemopneumothorax. Radiographic skeletal survey (Figure 1) showed a bilateral crescent fracture of the pelvis, left bone fractures of both forearms, and left ankle fracture. Computed tomography (CT) of the brain, chest, abdomen, spine, and pelvis showed right frontal lobe subarachnoid hemorrhage, sternal fracture with pneumomediastinum (surgical emphysema), right hemopneumothorax, bilateral subsegmental pulmonary embolism, bilateral lateral abdominal wall defects with underlying hematoma and emphysema that extended into the abdominal cavity extraperitoneally and retroperitoneally, right CIA dissection, comminuted fractures of the bilateral iliac crest bones, and bilateral crescent fracture-dislocation (Figures 2, 3).

**Table 1:** The laboratory results on admission, after irrigation and debridement, after laparotomy, and after relook laparotomy. ABG: arterial blood gas; I&D: irrigation and debridement; NA: not available

ABG	Admission	After I&D	After laparotomy	After relook laparotomy
pH	7.33	7.32	7.12	7.18
PaCO_2_ (mmHg)	32.3	32.3	48	57
PaO_2_ (mmHg)	136	140	278	204
HCO_3_ (mEq)	18.1	19	14.3	19
Base excess (mmol/L)	-8.0	-8.3	NA	NA
Hemoglobin (g/dl)	13.9	7.5	6.5	5.9
Lactate (mmol/L)	8.7	0.9	5.2	10.7

**Figure 4:**
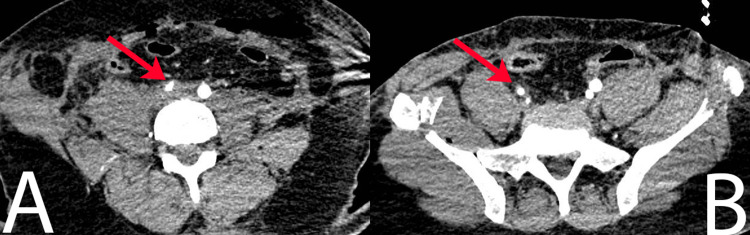
Axial CT images of the common iliac artery. (A) CT image demonstrating right common iliac artery near-total luminal occlusion caused by a thrombus. (B) CT image demonstrating attenuation of the right external and internal iliac arteries in comparison to the contralateral side. CT: computed tomography

The patient was immediately shifted to the Intensive Care Unit (ICU), and an urgent consultation with the vascular and orthopedics services was done. Additionally, he was started on traumatic brain injury protocol, heparin infusion protocol for pulmonary embolism, prophylactic antibiotics, sedative agents, continuous venovenous hemodiafiltration (CVVHDF), and low-dose inotropic support. Doppler ultrasound evaluation demonstrated biphasic popliteal, dorsalis pedis, peroneal arteries, and monophasic anterior tibial artery. The patient underwent computed tomography angiography (CTA) of the arterial tree of both lower limbs which revealed right CIA wall irregularity and interruptions with hypodense thrombus and near-total luminal occlusion 2 cm proximal to the bifurcation, with subsequent attenuation and narrowing of the right external, internal iliac, femoral, popliteal, and leg arteries (Figure 4).

**Figure 5:**
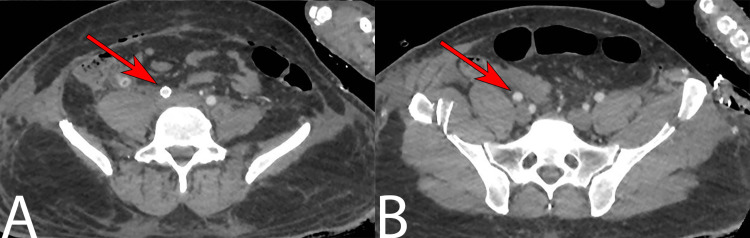
Axial CT images post-repair of the right common iliac artery. (A) Axial CT image demonstrating the stent that was placed in the right common iliac artery. (B) Axial CT image demonstrating patent right external and internal iliac arteries. CT: computed tomography

The patient was taken to the emergency operation room (OR) for micro-thrombectomy and stenting of the right CIA. Postoperatively, the right foot was warm with improved capillary refill and biphasic signals of the right lower limb arteries. Because the patient was in extremis, no further procedures were performed, and he was immediately shifted back to the ICU. In addition, the inotropic support was escalated, and he was started on heparin protocol for the vascular repair. Postoperative CT was performed which demonstrated patent arterial tree (Figure 5).

**Figure 6:**
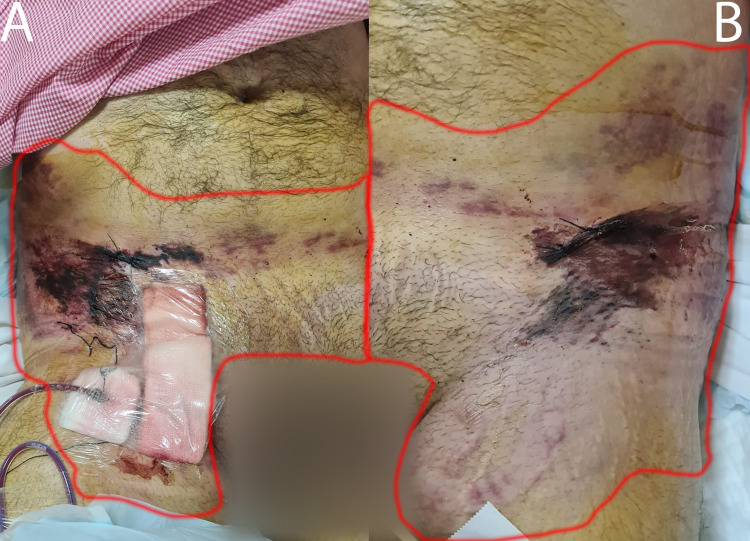
Clinical images of Morel-Lavallée lesion. (A) An image showing the degloving injury of the right entire lower abdominal wall extending to the upper thigh, flank, and the anterior abdomen. Blackish discoloration of the area surrounding the iliac crest overlying the open iliac wing fracture. (B) An image showing the degloving injury of the left lower abdominal wall extending to the upper thigh and flank. Similarly, blackish discoloration of the area surrounding the iliac crest can be seen with severe contusions over the flank, the upper thigh, and the anterior abdominal wall. Both wounds were communicating with each other underneath the skin.

The following day, a thorough re-assessment by the orthopedics team was performed which revealed a tense compartment of the right foot, which likely had developed secondary to revascularization after CIA repair, and blackish discoloration of the open pelvic wounds (Figure 6). The patient’s condition improved; therefore, he was taken to the OR for irrigation and debridement (I&D) of both open iliac wings fractures and MLLs, along with fasciotomy of the right foot compartments. The patient was placed in a supine position. The abdomen, pelvis and upper thighs, and right lower limb were prepped and draped in a sterile fashion. Fasciotomy of the foot was performed in a standard fashion utilizing three incisions. Attention was then shifted to the MLL. The stitches were removed and large amounts of hemoserous fluid were evacuated bilaterally (5 L in total). The skin edges were sharply debrided, and then a large curette was used to gently debride the dead fatty and subcutaneous tissues. Each wound was irrigated with 10 L of normal saline (NS). Strikingly, it was noticed that when fluid was flushed through one wound during the irrigation, it would come out of the other wound indicating severe degloving injury across the entire abdominal wall. Cultures were taken. Three drains were placed in, and the wounds were loosely sutured with non-absorbable monofilament sutures.

Postoperatively, the patient was shifted to the ICU as his condition was critical. In the first 24 hours, 400 mL of hemoserous fluid was drained. On the second-day post I&D, 900 mL of hemoserous fluid was drained from the left-sided drain. He had persistent readings of low BP despite efforts to raise BP with fluid management. His vital signs were BP of 98/61 mmHg, mean arterial pressure (MAP) 77 mmHg, and pulse rate 97 beats/minute. Arterial blood gas analysis was done and showed acceptable results (Table 1). Trauma, vascular, and orthopedic teams were consulted, and massive transfusion protocol (MTP) was activated for the transfusion of blood products. Examination of wounds showed minimal soakage with hemoserous fluid. The patient received two units of packed red blood cells, two units of fresh frozen plasma, and six units of cryoprecipitate. His vital signs improved. Subsequently, CTA was performed which showed no evidence of active extravasation indicating hemorrhage. Furthermore, the patient was diagnosed with acute kidney injury and rhabdomyolysis based on laboratory findings and was restarted on CVVHDF.

At the beginning of the second week, the patient‘s condition improved, and he was successfully extubated. He was taken for relook and I&D of the pelvic MLLs. The operation was carried out under general anesthesia in a supine position and similar steps were performed. This was followed by open reduction internal fixation of the left forearm fracture and left ankle fracture. At the end of the second week, his condition deteriorated as he became febrile (39°C) along with a drop in GCS requiring endotracheal intubation. Initially, septic screening was negative but eventually revealed right lung patchy infiltrates, and a positive endotracheal intubation culture of extended-spectrum β-lactamases *Escherichia coli*. The patient was started on antibiotics according to the culture sensitivity. The ICU team calculated the patient prognosis using the acute physiology and chronic health evaluation (APACHE) II score to predict hospital mortality, with a 73% estimated mortality, and the sequential organ failure assessment (SOFA) showed 95% estimated mortality. During the third week, the hospital course was turbulent as the patient developed sepsis and was mainly under ICU and trauma care.

At the end of the third week, chest radiographs delineated air under the diaphragm. Consequently, the patient underwent diagnostic laparoscopy. The abdominal cavity was found severely inflamed with extensive adhesions. During mobilization of the abdominal contents, bleeding was encountered from the splenic vessels that was difficult to control, thus, the procedure was converted to open exploratory laparotomy and splenectomy was performed. Examination of the lower abdominal and pelvic organs revealed spontaneous anterior rectal perforation. Postoperatively, the patient had refractory hypotension and became coagulopathic (Table 1). He underwent a relook laparotomy two times because of refractory hypotension. Despite maximum efforts to stabilize the patient, septic shock led to multiorgan failure and he passed away. Table 2 demonstrates a timeline of the main events.

**Table 2:** Timeline of the main events. ETT: endotracheal intubation; MTP: massive transfusion protocol; AKI: acute kidney injury; I&D: irrigation and debridement; CIA: common iliac artery; ORIF: open reduction internal fixation; GCS: Glasgow Coma Scale; EBAS: extended-spectrum β-lactamases Escherichia coli

Day	Event
0	Trauma day
1	Presentation to the emergency department
1	Thrombectomy and stenting of the right CIA
2	Right foot fasciotomy and I&D of Morel-Lavallée and open iliac wing fractures
4	Drop in hemoglobin and activation of MTP, rhabdomyolysis, AKI, re-initiation of continuous venovenous hemodiafiltration
6	Extubated
10	I&D of Morel-Lavallée lesion, and ORIF ankle and forearm fracture
12	Febrile, drop in GCS, and reintubated
13	Diagnosed with pneumonia
21	Rhabdomyolysis, AKI, re-initiation of continuous venovenous hemodiafiltration
23	Positive culture from ETT showing EBAS and sepsis
24	Spontaneous rectal perforation, air under diaphragm, diagnostic laparoscopy converted to exploratory laparotomy, and splenectomy
24	Drop in hemoglobin and activation of MTP
24	Relook laparotomy and refractory hypotension
25	Relook laparotomy, refractory hypotension, and coagulopathy
25	Death

## Discussion

Young et al. described a subtype of lateral compression injuries of the pelvis that is characterized by a variable disruption of the sacroiliac joint (SIJ) which extends to the posterior iliac wing and leaves a crescent-shaped segment of the ilium attached to the sacrum, hence the name [1]. Bilateral crescent fracture-dislocation is an extremely rare injury pattern. These injuries are largely surgical as they destabilize the pelvic ring. However, due to the severity of the multiple mortal injuries in our patient, the fracture was not surgically stabilized as he was in extremis. These injuries include bilateral crescent fracture-dislocation, bilateral open iliac wing fracture, CIA injury, and an extensive MLL. Thus, a damage control orthopedic approach must be sought when treating such injuries. It should be noted that the timing of the window of opportunity is from five to 14 days post-trauma. Definitive management should be carried out within this period. This window is followed by a period of immunosuppression during which all procedures must be delayed or avoided due to the risk of infection and multiorgan failure [5].

Examination of the pelvic area of our patient revealed a massive MLL that spanned the entire lower abdominal wall extending from the lower rib cage to the upper third of both thighs and involving both flanks. The presence of MLL has several implications on the management and surgical tactic [2]. Until now, there is no consensus regarding the management of MLL in the literature [3]. Management options include close observation with/without compression bandages, sclerodesis, percutaneous irrigation with closed suction drainage, open irrigation and debridement of the lesion, or aggressive debridement combined with excision of the lesion. In our case, observation was not a viable option due to the presence of bilateral open pelvic fractures. In addition, aggressive debridement would result in a massive soft-tissue defect; therefore, it was deemed inappropriate. Minimal invasive debridement with closed suction drainage has the advantage of the evacuation of the hematoma, providing drainage, reducing the bacterial burden, and preserving the soft-tissue envelope [6]. The presence of a large dead space poses substantial surgical difficulties. Several surgical methods have been reported attempting to limit fluid collection in the dead space [3]. In our experience, usage of quilting sutures combined with multiple closed suction drains ensures excellent control of the fluid collection and yields consistent outcomes. Quilting sutures are stitches that function to fix the overlying degloved subcutaneous tissue to the underlying fascia; therefore, it limits the available space for fluid collection. Bercial et al. studied three methods to reduce seroma formation in 40 abdominoplasty patients. Quilting sutures were found to result in significantly less fluid collection as measured by abdominal ultrasound [7]. A review of the literature failed to find a similar report of such extent. However, Daghmouri et al. reported a case of extensive MLL involving the lumbar spine and lower limbs, which complicated the patient’s condition and resulted in hemorrhagic shock [8]. Gummalla et al. also reported a case of extensive MLL which was missed on initial evaluation in a patient with a wrist fracture and abrasions over the trochanteric region. Two weeks later, the patient presented with progressive swelling over the hip region. Imaging studies revealed massive fluid collection above the fascia indicative of MLL [9]. In our case, the injury was obvious due to the presence of severe pelvic injury, bilateral open iliac wounds, and clinical examination that demonstrated flatulent collection in both flanks. However, MLL can easily be missed on initial assessment, as demonstrated by Gummalla et al. A high index of suspicion is crucial to diagnose such injuries. Nickerson et al. provided guidelines on the management of MLL. Their protocol depends on two main factors: skin viability and the volume of the aspirated fluid. Formal debridement is required when skin is not viable. Observation is chosen if the skin is viable provided that the aspirated fluid is ≤50 mL. In the case of aspirated fluid of ˃50 mL, I&D with suction drains is the treatment [10].

Blunt injury to the common iliac vessels is quite rare. Cestero et al. reported the incidence of blunt iliac arterial injuries (BIAI) in association with pelvic fractures to be 3.5%. Additionally, those vascular injuries were independently associated with emergency department hypotension, bowel injury, genitourinary injury, and Injury Severity Scale of ≥25. Interestingly, the incidence of bowel injury in the presence of BIAI was 24.9%, which is significantly higher without vascular injuries. The results of Cestero et al. demonstrated that patients with pelvic fractures and BIAI are more likely to die as the mortality rate in the group was 40.3% in comparison to patients without BIAI 15.4% [4].

## Conclusions

Crescent fracture-dislocation is an uncommon injury. Unfortunately, pelvic injuries are mostly associated with other injuries such as closed degloving soft tissue injuries, open fractures, and serious vascular injuries which significantly affect the management and the outcome and might lead to a turbulent hospital course. Prompt recognition and timely intervention are crucial to avoid morbidity and mortality.
